# Sleep disorders in children/adolescents with neurodevelopmental and neurological disorders: what evidences do we have with the use of non-pharmacological interventions?

**DOI:** 10.3389/frsle.2026.1758539

**Published:** 2026-03-18

**Authors:** Magda Lahorgue Nunes, Camila dos Santos El Halal

**Affiliations:** Pediatric Neurologist, Brain Institute (BraIns) – Pontifical Catholic University of Rio Grande do Sul, Porto Alegre, Brazil

**Keywords:** ADHD, adolescence, ASD, cerebral palsy, childhood, epilepsy, neurodevelopmental disorders, sleep

## Abstract

**Background:**

Sleep disturbances are highly prevalent across neurological and neurodevelopmental disorders (NDDs) and often exacerbate core symptoms, impair daytime functioning, and increase caregiver burden. Despite frequent clinical use of behavioral and educational strategies, the evidence base for non-pharmacological sleep interventions in this population remains scarce.

**Data source:**

This narrative review aimed to analyze behavioral interventions that can be used for sleep problems in children and adolescents with NDDs, and synthesizes data from recent studies that examined those non-pharmacological interventions in epilepsy, autism spectrum disorder (ASD), attention-deficit/hyperactivity disorder (ADHD), cerebral palsy (CP), and rare genetic neurodevelopmental conditions (RGNCs).

**Results:**

Across NDDs, insomnia symptoms are highly prevalent, with circadian disturbances and sleep-disordered breathing also common in some groups. Behavioral and parent-led interventions—including psychoeducation, sleep hygiene, structured routines, and extinction-based strategies—consistently improve parent-reported sleep and often enhance daytime behavior, though objective sleep gains are smaller. In epilepsy and ASD, tailored behavioral–educational programmes are both effective and acceptable. In ADHD, behavioral sleep interventions and melatonin improve sleep, with behavioral approaches also yielding modest reductions in ADHD symptoms. Evidence for CP and RGNCs is limited but supports individualized, multimodal management targeting both behavioral and physiological contributors, while syndrome-specific chronobiological treatments offer only partial benefit.

**Conclusions:**

Behavioral and educational sleep interventions are generally safe, acceptable, and clinically useful across NDDs, particularly when embedded in multidisciplinary, condition-informed care. However, their efficacy is constrained by small, heterogeneous trials and non-standardized outcome measures. Robust, syndrome-specific randomized studies with harmonized sleep and daytime outcomes are urgently needed to guide evidence-based practice.

## 1 Introduction

Neurodevelopmental disorders (NDD) are a group of conditions that often manifest during the developmental period and are the result of developmental deficits of the central nervous system that impact one or more areas of functioning. NDD produce impairments across social, academic, personal, or occupational functioning (Rigney et al., 2018).

Sleep is essential for brain development and overall health, particularly in children with NDD and evidence suggest that children with NDD have higher rates of sleep problems and may be more vulnerable to the impact of poor sleep than their typically developing peers. Sleep disruptions can impact brain structure and function and might lead to dysfunction of neurotransmitter systems, metabolism, hormonal balance and inflammatory processes. Poor sleep may result in increased symptom presentation of the NDD (Bruni et al., 2025; El Halal and Nunes, 2025).

The pathophysiology of sleep disorders in children/adolescents with NDD is linked to genetic and epigenetic factors that influence endogenous dysfunction in the release of hormones, neurotransmitters and perception of zeitgeber. Understanding the complex relationship between sleep patterns and NDDs is essential for developing targeted interventions and enhancing the overall quality of life for affected individuals and their families (Bruni et al., 2025).

The combination of pharmacological and non-pharmacological strategies (sleep hygiene, parent education, adapted intervention based on cognitive behavioral therapy) generally lead to broad improvements on symptoms. However, the assessment and management of sleep problems should involve a multidisciplinary team, because of the high heterogeneity in etiologies, clinical presentations and comorbidities of NDDs (Rigney et al., 2018; Bruni et al., 2025; Phillips et al., 2020).

The aims of this narrative review are to describe the most common sleep disorders and complains in children and adolescents with NDD and neurological disorders and to analyze behavioral interventions that can be used in this population.

## 2 Methodology

We have performed a narrative review of the literature in the topic sleep interventions in children with neurological disorders and NDD. The combination of terms used to search articles were “sleep disorders,” “neurodevelopmental disorders,” “autism spectrum disorders,” “attention deficit disorder,” “epilepsy,” “cerebral palsy” “genetic disorders,” and “behavioral therapy.” Our focus was on non-pharmacological interventions. However, we have also included articles that mentioned the use of melatonin. The age range of the population selected was specifically children and adolescents (< 19 years). Articles that have not approached sleep interventions or that involved adult population were excluded.

## 3 Results

A total of 44 articles were selected to support this review, among them systematic reviews (*n* = 27), randomized controlled trials (*n* = 5), prospective single-case and cohort studies (*n* = 11) and one guideline from the American Academy of Neurology. The age range of participants cited in the articles varied between 2 and 18 years. In order to give a more comprehensive reading we have divided articles in different topics, starting with the description of cognitive and behavioral interventions used for sleep complains, followed by studies that have evaluated sleep in mixed NDDs and neurological disorders and studies with focus on specific NDDs or Neurological disorders such as autism spectrum disorders, attention deficit disorder, epilepsy, cerebral palsy, and more rare genetic conditions.

### 3.1 Description of cognitive and behavioral interventions used for sleep complains

Cognitive and behavioral interventions have been developed to approach behavioral sleep problems in school aged children and are commonly used in clinical practice Rigney et al., (2018). The most frequent ones used in the pediatric population are described below and in Table 1.

**Table 1:** Behavioral interventions for sleep disorders in children.

**Type of intervention**	**Brief description of intervention**
Unmodified extinction	The infant is placed in bed while awake, left alone until asleep, and night waking is ignored. The infant learns to self-soothe once realizing that nighttime crying does not result in parental attention.
Extinction with parent presence	The parent remains in the room during extinction, acting as a reassurance for the child but providing little interaction.
Graduated extinction	Involves ignoring negative behaviors (i.e., crying) for a given amount of time before checking on the child. The parent gradually increases the amount of time between crying and parental response. Parents provide reassurance through their presence for short durations and with minimal interaction
Extinction (planned ignoring)	Standard withholding rewards (such as TV) and ignoring sleep disruptive behavior, gradually increasing the time before the child is attended.
Gradual distancing (stimulus fading)	Parent gradually increases the distance from the child
Bedtime fading	Involves delaying bedtime closer to the child's target bedtime. The goal of this treatment is for the child to develop a positive association between being in bed and falling asleep rapidly. Bedtimes can be gradually moved earlier.
Sleep scheduling or Scheduled awakening	Scheduling regular, appropriate sleep and wake times that allow for an adequate sleep opportunity. Parents wake up the child 30 min before the time he/she usually awakens spontaneously.
Sleep restriction	Reducing total sleep time to 90% of average night-time sleep while keeping a consistent schedule. Once behaviors improve, fade back total amount of sleep to age-appropriate level
Bed pass	Give the child a special card good for one free trip out of their room each night or one visit from a parent. When the child uses the pass, the card is surrendered for the rest of the night and if they leave the room again that night, they are walked back to their room with no words or attention. The child can trade unused passes for a reward at the end of the week.

Modified from Rigney et al. (2018) and Johnson and Zarrinnegar (2021).

Psychoeducation: this approach can be provided to the children/adolescent and/or parents and focuses on sleep and sleep hygiene, as well as relaxation strategies. Sleep hygiene incorporates a variety of behavioral strategies that seek to create associations between the bed and sleep (e.g., using the bed only for sleeping) and to reduce the presence of antecedents that may interfere with sleep, such as caffeine and digital media use. Parent education, as teaching parents the basics of childhood sleep, sleep hygiene, how to establish consistent and sleep-inducing bedtime, and how to implement behavioral interventions. It impacts on sleep latency, child and family functioning (Rigney et al., 2018).

**Figure 1:**
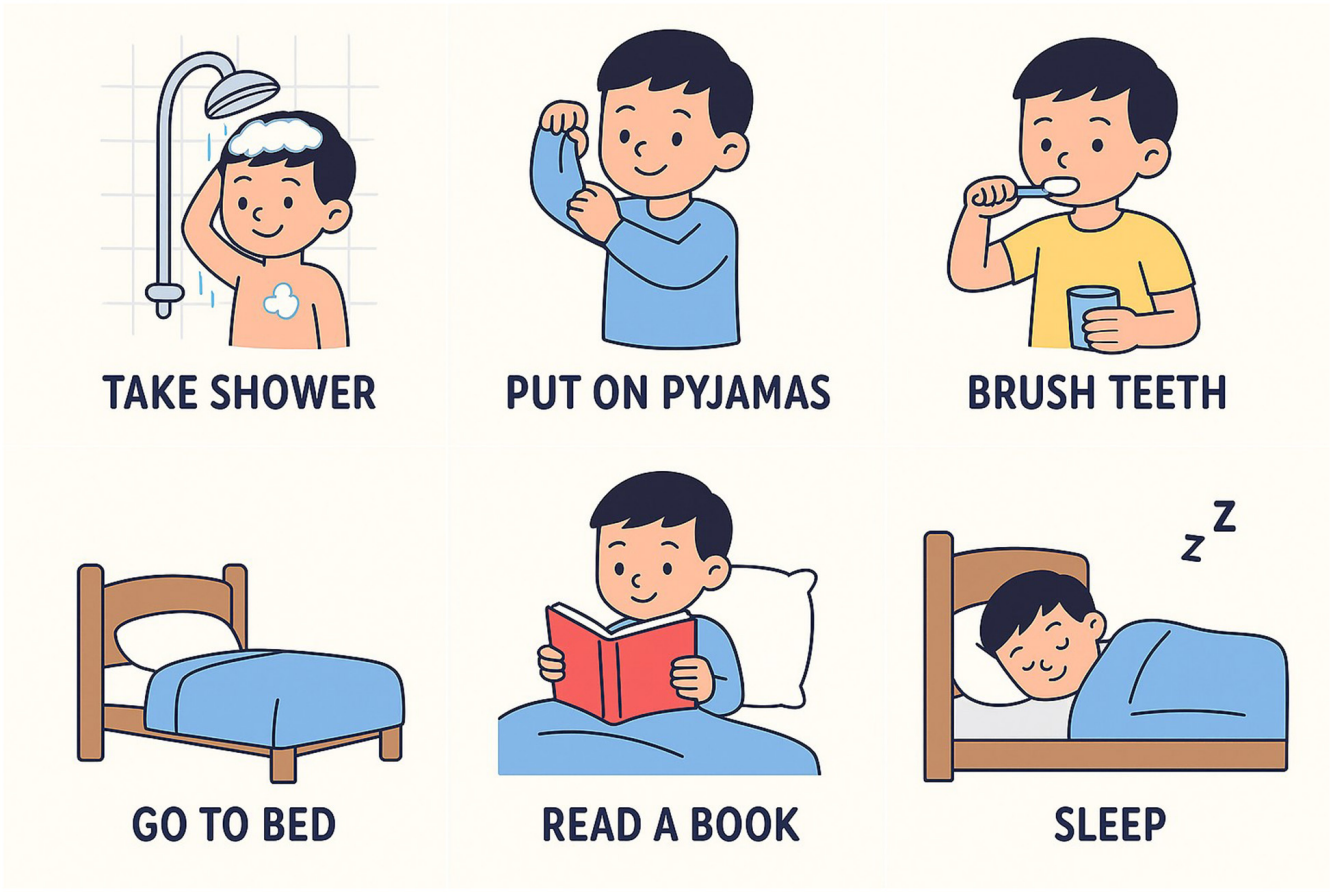
Practical tips for parents regarding sleep hygiene.

Positive bedtimes routines: implementation of consistent series of activities that help the child with transition to sleep (i.e., clean up toys, brush teeth, go to the toilet, put pajamas on, read books, get in bed, lights off) Impact on bedtime difficulties, settling problems; usually combined with other techniques (Johnson and Zarrinnegar, 2021). For children with NDD, these can be implemented in the form of a personalized visual routine as shown in Figure 1.

Cognitive strategies: are used to address non-productive beliefs about sleep, including the belief that the child cannot change his/her sleep difficulty. Coping strategies are also included (e.g., relaxation skills such as abdominal breathing) (Rigney et al., 2018).

Cognitive-behavioral therapy (CBT-I): widely used and well established as an effective treatment for adults. The approach can be adapted for adolescents, combining behavioral components—such as sleep restriction and the development of healthy sleep habits—with cognitive strategies aimed at reframing unhelpful beliefs, expectations, and worries about sleep (Johnson and Zarrinnegar, 2021).

Bright light therapy: used to treat insomnia as well as delayed sleep phase disorder mostly in adults. Using bright light immediately upon waking can advance one's sleep cycle, increasing the likelihood of earlier sleepiness (Bourchtein et al., 2020).

Imagery rehearsal therapy (IRT): commonly used and effective as treatment for reducing frequency and intensity of nightmares in adults. It comprises two main components: psychoeducation about the link between daily experiences and imagery in nightmares, and modification of the nightmare to be more pleasant with imagery practiced in a relaxed state. Efficacy in children has not been well studied (Bourchtein et al., 2020).

Chronotherapy: Bedtime and wake time are gradually shifted later each day until the child aligns with a more typical sleep schedule, a strategy particularly useful for circadian rhythm sleep–wake disorders, especially the delayed sleep–wake phase type (Johnson and Zarrinnegar, 2021).

### 3.2 Studies that evaluated mixed NDDs and neurological disorders

Bourchtein and collaborators developed a systematic review to evaluate pediatric nonpharmacological sleep interventions co-occurring in population with mental health conditions (Bourchtein et al., 2020). Twenty studies were included in the qualitative synthesis. For insomnia the treatment involved psychoeducation and/or sleep hygiene, RCTs observed that adolescents receiving the treatment intervention experienced greater objectively assessed total sleep time and earlier bedtime but no improvement on sleep efficiency. One study focused exclusively on circadian rhythm sleep-wake disorders found that the combination of bright light therapy with CBT led to significant improvements on several sleep parameters, including advances in sleep onset. IRT was effective in reducing frequency of nightmares in different populations and age ranges. Youth with comorbid externalizing problems (i.e., ADHD/behavioral difficulties) are likely to benefit from the incorporation of aspects of behavioral parent training into sleep interventions. For youth with internalizing problems, there is currently little support for using multimodal sleep or CBT-I interventions alone, and the addition of a CBT component that focuses specifically on the internalizing problems may be necessary.

Kamara and collaborators developed a systematic review and meta-analysis of behavioral interventions for sleep disruption in pediatric neurodevelopmental and medical conditions (Kamara et al., 2025). The most studied neurodevelopmental and medical conditions were ADHD, ASD, and Down syndrome. Data from 15 RCTs were examined (1,374 participants, 78% male, 71% White). Sleep disruptions were predominantly insomnia symptoms. Intervention content included parent training, sleep hygiene education, and relaxation strategies. Adaptations to the interventions for use in children with neurodevelopmental and/or medical conditions included behavioral strategies commonly used in those conditions, sleep education specific to the condition, and/or use of case examples specific to the condition. No studies reported on adverse effects. Behavioral sleep interventions had a significant effect on sleep satisfaction, bedtime resistance, and ADHD symptoms at postintervention. At follow-up, effects were maintained only for sleep satisfaction. Parent rating of child sleep duration improved at follow-up but not postintervention. In this systematic review of children with pediatric neurodevelopmental and medical conditions, behavioral sleep interventions led to improvements in some domains of sleep health, most notably sleep satisfaction and quality, as well as some daytime condition-related symptoms. Sleep duration, as measured with actigraphy, did not improve.

Rigney and collaborators developed a systematic review to explore the feasibility of a behavioral sleep intervention for insomnia in children with neurodevelopmental disorders (Rigney et al., 2018). They have evaluated the methodological quality of parent-delivered behavioral sleep interventions for children with NDD, specifically Attention-Deficit/Hyperactivity Disorder, Autism Spectrum Disorder, Cerebral Palsy, and Fetal Alcohol Spectrum Disorder. The most frequently reported sleep symptom included bedtime resistance, night-waking, early morning awakening, and co-sleeping. The most common interventions used were implementation of healthy sleep practices, reinforcement, graduated extinction, and faded bedtime.

Although recent studies that evaluated the behavioral treatments for sleep disorders in NDD reported a moderate-to low level of evidence that supports these interventions, as only 25% of individuals responded and no changes were obtained in sleep measures, sleep hygiene education improved daytime behaviors, quality of life and sense of competence in parents (Rigney et al., 2018; Bourchtein et al., 2020; Kamara et al., 2025).

## 4 Studies with focus on specific NDDs or neurological disorders

### 4.1 Epilepsy

Sleep disturbance is a surprisingly common comorbidity for children with epilepsy (CWE) with sleep problems occurring more often than in typically developing (TD) children (Malow and Vaughn, 2002). Even in the absence of nocturnal seizures, sleep problems as reported by parents are 12 times more common in 4–10-year-old CWE than in children without epilepsy (Gutter et al., 2013).

In the study by Cook and collaborators a small sample of parents of children with epilepsy were interviewed and made considerations for the content and delivery of behavioral sleep interventions (Cook et al., 2021). Among the many suggestions parents wanted to be presented with were a range of sleep management options from which they could select, personalized information and suggestions for behavior-change options, help in addressing child anxiety around sleep, general educational information about sleep and the relationship between sleep and epilepsy and to receive help, support, and reassurance around children's sleep.

The CASTLE programme was developed by Wiggs and collaborators and preliminarily evaluated COSI, an online behavioral sleep intervention tailored for parents of children with epilepsy (CWE) (Wiggs et al., 2021). Recognizing that many sleep difficulties in CWE share behavioral origins with those seen in typically developing children, the authors adapted two evidence-based pediatric behavioral sleep interventions and integrated epilepsy-specific content derived from interviews with parents and children. The resulting programme includes personalized modules, embedded parent and child experiences, sleep–seizure education, strategies for common sleep problems, and guidance for managing sleep-related anxieties. In a two-phase process, parents reviewed COSI's content and functionality, reporting universal acceptability and usability, with all indicating they would recommend it to other families. This work highlights the value of co-creation with families to ensure relevance and engagement and sets the stage for testing COSI's clinical effectiveness in the CASTLE Sleep-E randomized controlled trial.

In a clinic-based randomized controlled trial evaluating a behavioral–educational sleep intervention for pediatric epilepsy, 100 toddlers and preschool-aged children with epilepsy were randomized to receive either a structured behavioral sleep intervention or attention-matched usual care, with outcomes assessed over a 12-month period using actigraphy and standardized questionnaires (Tsai et al., 2020). Children in the intervention arm received standard neurology care plus a three-session behavioral–educational sleep program, consisting of individualized actigraphy-based feedback, structured sleep hygiene education, and tailored goal setting, delivered at baseline and reinforced through booster sessions at 3 and 6 months, with an additional follow-up phone call at 9 months. Across sessions, parents were guided in applying five core sleep-promoting strategies (recognizing the importance of sleep and the recommendations for age-appropriate sleep durations with a bedtime before 9 PM; establishing adequate sleep habits and consistent bedtime routines as well as sleep schedules; minimizing screen time < 2 h each day and keeping the bedroom dark, cool, and media free; avoiding caffeine consumption and long naps after 3 pm; and actively engaging in > 1 h of physical activity suitable to the child's motor and developmental capabilities), addressing routines, schedules, environment, activity, and barriers to behavioral change. The intervention group demonstrated significantly greater improvement in nocturnal sleep efficiency (adjusted mean difference 2.03%, 95% CI 0.20–3.86; *P* = 0.03) and longer total nighttime sleep (adjusted mean difference 16.13 min, 95% CI 0.24–32.03; *P* = 0.04) compared with usual care. No significant effects were observed for maternal sleep knowledge or maternal sleep quality. These findings indicate that integrating a structured sleep intervention into routine neurology visits yields measurable and durable improvements in sleep among young children with epilepsy.

### 4.2 Autism Spectrum Disorder

Sleep disturbances, most commonly insomnia, represent a significant component of Autism Spectrum Disorder (ASD) occurring in around 80% of the affected children and adolescents (Devnani and Hegde, 2015). Sleep problems are dimensionally associated with the severity of ASD symptoms and as well as with daytime struggles. Studies show a link between specific genetic variants, such as 22q11.2, and sleep disturbances in ASD, suggesting that the sleep problems are not merely behavioral. Sleep disorders have been linked to poorer executive functioning in children with ASD, underscoring the impact that sleep disruption can have on cognitive development and performance (Singh and Zimmerman, 2023).

In a study designed to evaluate the effectiveness of a brief, parent-delivered behavioral sleep education program for young children with ASD who exhibited significant sleep difficulties, parents in the intervention group received targeted training in sleep hygiene, bedtime routines, behavioral strategies for managing sleep-onset delay and night wakings, and methods for reducing inappropriate sleep associations, while the control group received standard care. The program produced significant improvements in children's sleep, including reduced sleep-onset latency, fewer and shorter night wakings, longer total sleep duration, and better overall sleep quality, as measured by parent report and actigraphy. Additionally, improvements were observed in daytime behavior and parental stress, suggesting both direct and indirect benefits of sleep enhancement. The findings demonstrate that a structured parent-based behavioral intervention is a feasible, low-cost, and effective approach for addressing sleep problems in children with ASD (Malow et al., 2014).

Cortesi and collaborators studied 166 children with ASD, with sleep onset insomnia and impaired sleep maintenance, the age range of the population was 4–10 years, and they were assigned randomly to either (1) combination of controlled-release melatonin and cognitive–behavioral therapy; (2) controlled-release melatonin; (3) four sessions of cognitive–behavioral therapy; or (4) placebo drug treatment condition. The active treatment groups all resulted in improvements across all outcome measures, with moderate-to-large effect sizes from baseline to a 12-week assessment. Melatonin treatment was mainly effective in reducing insomnia symptoms, while cognitive–behavioral therapy had a light positive impact mainly on sleep latency, suggesting that some behavioral aspects might play a role in determining initial insomnia. The combination treatment group showed a greater proportion of treatment responders achieving clinically significant changes. They concluded that adding behavioral intervention to melatonin treatment seems to result in a better treatment response for those population (Cortesi et al., 2012).

Behavioral interventions are considered first-line of treatment by the American Academy of Neurology for sleep disorders in children and adolescents with ASD, after addressing coexisting medical conditions- such as intellectual disability, sleep apnea, epilepsy, gastrointestinal disturbances, mood disorders, and Attention-Deficit and Hyperactivity Disorder—and medication use—antiseizure medications, stimulants, or psychotropics (Williams Buckley et al., 2020). The guidelines also stress that, although there is no current evidence to support the use of weighted blankets, their usage may be useful for some individuals. Melatonin should be considered as a treatment option once behavioral strategies have failed to significantly improve sleep.

### 4.3 Attention deficit hyperactivity disorder

Chaulagain and collaborators presented a systematic meta-review of systematic reviews on attention deficit hyperactivity disorder (ADHD), among the findings they reported that ADHD was associated with a high degree of comorbid somatic conditions (e.g., obesity, asthma, headache/migraine, sleep problems) and psychiatric disorders (e.g., other neurodevelopmental, affective, anxiety, and eating disorders) (Chaulagain et al., 2023). However, the analyzed studies were mostly correlational and lacking data on temporality. The reviews do not sufficiently distinguish comorbidities from side effects of medication. The causal mechanisms in the comorbidities were not addressed. The studies revealed impaired sleep among children with ADHD, including those measured by polysomnography or actigraphy. The most significant findings were the association of short sleep duration with hyperactivity, and lighter sleep when compared to those without ADHD, higher sleep latency and lower sleep efficiency. Shorter sleep time correlated with more severe ADHD symptoms (Singh and Zimmerman, 2023).

In a randomized controlled trial, the Hiscock et al. evaluated whether a brief behavioral sleep intervention could improve sleep and ADHD-related outcomes in 244 children aged 5–12 years with ADHD and moderate to severe sleep problems (Hiscock et al., 2015). Children were randomized to receive two sleep-focused behavioral consultations plus a follow-up phone call or to continue usual care, with outcomes assessed at baseline and at 3 and 6 months using validated parent and teacher questionnaires, direct working-memory testing, and actigraphy in a subsample. The intervention led to modest but significant reductions in ADHD symptom severity at both follow-ups, along with substantial improvements in sleep problems, behavior, daily functioning, and psychosocial quality of life; teachers also noted better behavior, and working memory was slightly improved at 6 months. Mediation analyses indicated that one-third to one-half of the improvement in ADHD symptoms was attributable to improved sleep. Although parent mental health did not change, parents reported fewer missed or late workdays at 3 months, supporting the broader functional benefits of this brief, scalable intervention.

In the systematic review conducted by Larsson et al. (2023), the effectiveness of sleep interventions for children and adolescents aged 6–18 years with ADHD was evaluated. The included studies assessed both behavioral sleep interventions—which incorporated structured sleep hygiene education, individualized behavioral strategies targeting specific sleep problems, and parent-focused support—and pharmacological treatments, namely melatonin (3–6 mg fast-release), and eszopiclone (2–3 mg). Although the overall certainty of evidence was low and the studies were heterogeneous, behavioral interventions produced the most consistent benefits, yielding moderate improvements in parent-reported sleep disturbances and small but meaningful short-term reductions in ADHD symptoms. For sleep disturbances, the effect size was moderate for behavioral interventions among children aged 3–13 years. Melatonin was associated with objective improvements in total sleep time, sleep onset latency, and sleep efficiency, whereas eszopiclone demonstrated minimal effects on sleep outcomes. Therefore, the findings indicate that behavioral sleep interventions and, to a lesser extent, melatonin may improve sleep in youth with ADHD.

### 4.4 Cerebral palsy

Sleep disturbances occur in over a quarter of children diagnosed with cerebral palsy (CP) (Nadeem et al., 2025). Disorders of sleep initiation and maintenance were the most prevalent (28%), followed by sleep-wake transition disorders (19%), and sleep breathing disorders in 17%. Abnormal sleep scores have been observed across various cerebral palsy subtypes, with higher rates generally seen in children with bilateral spastic involvement, followed by those with dyskinetic presentations (Newman et al., 2006). Furthermore, a study by Newman et al. found that disorders of initiation and maintenance of sleep were more frequent in children with spastic quadriplegia (OR = 12.9, 95% CI 1.9–88.0) or dyskinetic CP (OR = 20.6, 95% CI 3.1–135.0) (Nadeem et al., 2025). Similarly, sleep disturbances increase in parallel with motor impairment severity: while approximately 15% of children at GMFCS level I exhibit abnormal sleep patterns, this proportion rises to about 50% among those at level V (Horwood et al., 2019).

**Figure 2:**
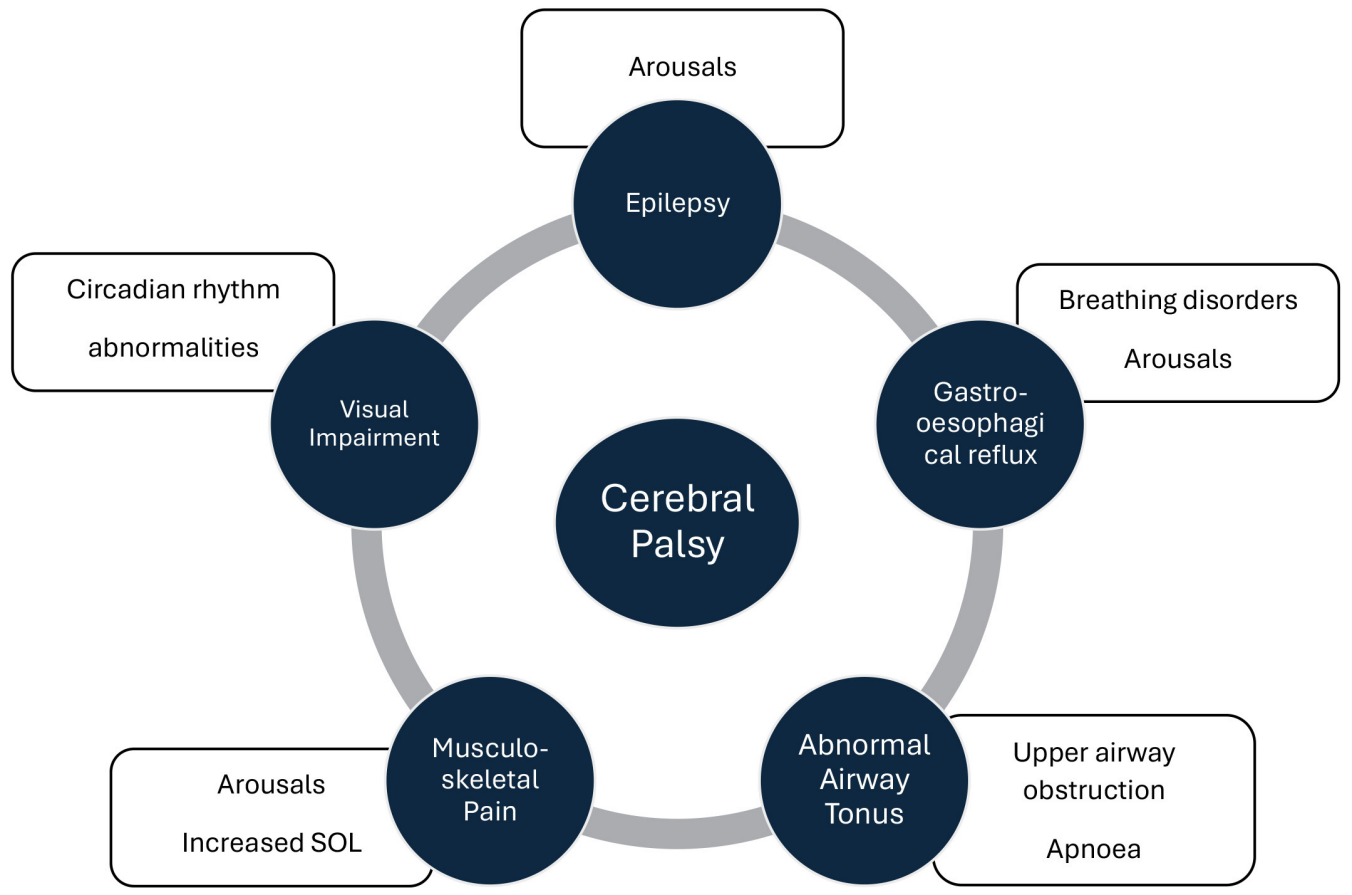
Sleep abnormalities associated with cerebral palsy comorbidities. SOL, Sleep Onset Latency.

Comorbidities -medical and environmental—appear to further shape the risk profile for sleep disturbances in children with CP (Figure 2). Newman and cols found that active epilepsy showed a strong association with the presence of a sleep disorder (OR 17.1, 95% CI 2.5–115.3), as did living in a single-parent household (OR 3.9, 95% CI 1.3–11.6) (Newman et al., 2006). Pain arising from spasticity, dystonia, orthopedic deformities (scoliosis, hip subluxation), and muscle spasms is commonly an underrecognized driver of nocturnal awakenings and poor sleep quality (Angriman et al., 2015). Notably, interventions that alleviate these sources of discomfort—such as intrathecal baclofen—have been associated with reductions in night wakings (Ramstad et al., 2010). Botulinum toxin A has also been reported to improve sleep onset and reduced nighttime waking (Li et al., 2025).

Besides disorders of initiation and maintenance of sleep, children with CP have an increased prevalence of obstructive sleep apnoea (OSA). In a multicentre cross-sectional study of 312 Brazilian children with cerebral palsy (median age 6 years), the overall prevalence of high risk for obstructive sleep apnoea syndrome) was 9%. Overall, children with more severe motor impairment (GMFCS V) exhibited a substantially greater risk profile for OSAS than those with milder CP or typical development (Dias et al., 2025). Physiopathologically, OSA results from recurrent upper airway obstruction during sleep and children with CP, often present additional anatomic and neuromuscular factors—such as midface disproportion, mandibular alterations, and abnormalities in upper airway tone (hypotonia, hypertonia, or dystonia)—that further compromise airway stability. Other contributors include impaired central respiratory control, obesity, and medications that depress upper-airway muscle activity (Simard-Tremblay et al., 2011). The subsequent sleep fragmentation, chronic nocturnal hypoxemia, and hypoventilation contribute to a significant risk of neurobehavioral, psychiatric, and cardiopulmonary complications in these children (End et al., 2022). Adenotonsillectomy (AT) is considered first-line treatment for moderate to severe OSA in children, and this approach has been extended to the management of OSA in children with CP as a strategy to avoid tracheotomy (Hsiao and Nixon, 2008). Children with CP who received OSA treatment demonstrated significant improvements in sleep disturbance, daytime functioning, and caregiver concern, alongside an 18% mean increase in parental quality-of-life scores relative to untreated controls (Hutson and Snow, 2020).

Epilepsy is a common comorbidity in children with cerebral palsy, with prevalence estimates ranging from approximately 15% to 55%. In this population, the presence of epilepsy appears to further heighten vulnerability to sleep disturbances; however, the underlying mechanisms remain incompletely defined. Proposed contributors include epilepsy-related alterations in endogenous sleep regulation, sleep fragmentation secondary to nocturnal seizures and associated arousals, and the potential impact of antiseizure medications on sleep architecture (Simard-Tremblay et al., 2011). In a systematic review, the association between sleep and epilepsy in children with CP was more strongly associated to sleep problems when epilepsy was active (34.1% among children with active epilepsy vs. 18.2% among those with controlled epilepsy, and 12.7% among those without epilepsy), and similar trends were found among preschoolers. Among sleep problems, disorders of initiation and maintenance of sleep were found among 36.6% of children with active epilepsy, in contrast to 13.6% of those with controlled epilepsy and 17.6% of children without epilepsy. Those with active epilepsy showed higher risk of disorders of excessive somnolence (24.4%, in contrast with 13.6% and 7.8% of those with controlled and without epilepsy) and sleep-wake transition disorders (22%, 22.7%, and 10.8% of children with active, controlled, or without epilepsy, respectively). Nighttime awakening was found in 32.3% of children with cerebral palsy and epilepsy. Sleep disordered breathing was also more prevalent in children with epilepsy (58%), even when compared to groups of children with other NDDs but neither cerebral palsy or epilepsy (Horwood et al., 2019).

Cortical visual impairment affects an estimated 20% to 50% of children with cerebral palsy. Because light signals transmitted through the retinohypothalamic pathway play a central role in activating the suprachiasmatic nuclei—the master regulator of circadian rhythms—disruptions in visual processing can significantly alter circadian entrainment. Melatonin, a key hormone governing the sleep–wake cycle, is released by the pineal gland in darkness and inhibited by light exposure (Simard-Tremblay et al., 2011). Notably, children with severe visual impairment have a substantially higher likelihood of experiencing sleep disturbances (OR = 12.5, 95% CI 2.5–63.1) (Newman et al., 2006).

Given the multifactorial nature of sleep disturbances in children with CP—including contributions from motor impairment, pain, epilepsy, respiratory dysfunction, and sensory comorbidities such as visual impairment—accurate evaluation requires a structured and multimodal approach. Clinical history and caregiver report are essential, but recent evidence highlights important gaps in the sensitivity and specificity of commonly used tools in children with severe motor impairment. In a scoping review examining sleep outcome measures used in postural care interventions for children with severe CP (GMFCS IV–V), Hutson and Snow found that no single instrument adequately captured the full spectrum of sleep disturbances relevant to this population. Instead, the combination of a caregiver-reported questionnaire—particularly the *Sleep Disturbance Scale for Children* (SDSC), which covers multiple sleep domains—and actigraphy, which provides objective estimates of sleep–wake patterns and nocturnal movement, emerged as the most suitable strategy for routine assessment (Hutson and Snow, 2020). This paired approach allows both subjective symptoms (e.g., nighttime discomfort, settling difficulties, awakenings) and objective sleep fragmentation to be monitored over time, offering a more reliable framework for evaluating the impact of interventions such as tone management, respiratory treatment, pain control, and circadian-rhythm support in visually impaired children.

Literature consistently shows a significant lack of high-quality evidence supporting non-pharmacological interventions in cerebral palsy, since most available studies are observational, small, or methodologically limited, with very few randomized controlled trials or long-term evaluations (Almeida et al., 2024). Vershuren et al, therefore, emphasize a multimodal approach, consisting of initially first identifying and treating comorbidities that disrupt sleep—such as pain, spasticity, reflux, anxiety, ADHD, or poorly controlled seizures—followed by evaluation for primary sleep disorders, particularly sleep-related breathing disturbances. When circadian dysregulation is suspected, strategies like consistent schedules, morning light exposure, evening light restriction, and, when appropriate, timed melatonin may support entrainment. Behavioral sleep interventions are commonly recommended, although evidence in CP is largely extrapolated from studies in typically developing children and those with other neurodevelopmental disorders. Pharmacotherapy is generally reserved for refractory cases; melatonin has the most support, while other agents (e.g., gabapentin, tricyclic antidepressants, clonidine) are used cautiously given limited empirical data and potential sleep-worsening effects of some medications (Verschuren et al., 2017).

### 4.5 Rare genetic neurodevelopmental conditions

Rare genetic neurodevelopmental conditions (RGNC) comprise a group of heterogeneous conditions resulting from chromosomal alterations, present in fewer than one in 2000 individuals (Woodford et al., 2025). Sleep disturbances are highly prevalent across this group of individuals (up to 90% of prevalence) and represent a major contributor to impaired daytime functioning, behavioral dysregulation, and caregiver burden. Although these syndromes share broad clinical features such as intellectual disability and behavioral challenges, the underlying mechanisms of sleep disruption may vary significantly, ranging from circadian dysregulation to impaired ventilatory control, which may warrant tailoring behavioral and educational interventions to each syndrome's specific sleep phenotype (McLay et al., 2019).

In a single-case multiple-baseline study conducted by Woodford et al, the efficacy and acceptability of function-based behavioral sleep interventions in eight children with RGNC (mean age 7.3 years) were evaluated. Intervention components were introduced sequentially across up to three phases, progressing from minimally to more restrictive strategies to determine the least intensive approach required to achieve clinically meaningful change. The interventions led to improvements in sleep onset latency (7/7 participants), night wakings (6/7), early morning waking (3/3), and unwanted bedsharing (3/3). For most children, clinically relevant gains occurred during the less restrictive intervention phases—specifically circadian adjustments, antecedent modifications, and reinforcement-based procedures. Nevertheless, modified extinction strategies were required for five participants. Treatment effects were maintained at follow-up, which happened between 10 and 14 weeks after the interventions, and parents rated all procedures as acceptable (Woodford et al., 2025).

In a systematic review of nine behavioral intervention studies involving children with various RGNC, treatment targets included sleep-onset delay, bedtime resistance, frequent or prolonged nocturnal awakenings, attention-seeking behaviors directed toward caregivers, co-sleeping, early morning awakenings, and nocturnal crying or screaming (McLay et al., 2019). Across studies, interventions were predominantly multicomponent and grounded in sleep-hygiene principles, emphasizing consistent bedtime routines and a sleep-conducive environment with appropriate temperature control and minimal auditory or visual stimulation. Core behavioral components included sleep–wake schedule adjustments, bedtime fading—with or without response-cost procedures—extinction or modified extinction strategies, and reinforcement-based approaches. Bedtime fading, implemented by delaying sleep onset and gradually advancing bedtime, was particularly effective, and when combined with response cost produced greater improvements than bedtime scheduling alone. Extinction techniques varied in restrictiveness, while reinforcement typically involved providing rewards for successful night-time sleep. For anxiety-related sleep disturbances, desensitization paired with differential reinforcement was used. Parent-training programs delivered either in person or through written materials supported caregivers in applying sleep hygiene, reinforcement, extinction, routine establishment, and antecedent–consequence strategies. Across studies, these interventions generally improved sleep-onset latency, bedtime resistance, nocturnal awakenings, co-sleeping, and disruptive night-time behaviors, with bedtime fading combined with response cost showing particularly strong effects. Parent training—whether in person or via written materials—also led to measurable gains. However, the overall certainty of evidence is limited, as only three studies met criteria for adequate methodological rigor (McLay et al., 2019).

#### 4.5.1 Smith–Magenis syndrome

Smith–Magenis syndrome (SMS) is characterized by severe sleep–wake disturbances, which significantly intensify behavioral dysregulation. Sleep problems include difficulties initiating and maintaining sleep, frequent nocturnal awakenings, early sleep onset and early morning waking, nighttime agitation, excessive daytime sleepiness, and in some cases absence of REM sleep. The hallmark biological abnormality is a complete inversion of the circadian melatonin rhythm, with melatonin secretion peaking around midday instead of nighttime; this pattern is consistently documented in SMS individuals through plasma and urinary assays. The circadian dysregulation is attributed to RAI1 haploinsufficiency, which disrupts the transcriptional regulation of key circadian clock genes—including *CLOCK, PER1, PER2*, and *BMAL1*-among others- gene regulation and —as well as pathways involved in light-mediated entrainment (Kaplan et al., 2020; Poisson et al., 2015).

Effective treatment centers on correcting the inverted melatonin rhythm. The recommended pharmacological strategy is morning β-1 adrenergic antagonists to suppress abnormal daytime melatonin, combined with evening prolonged-release melatonin (2–6 mg) to restore night-time secretion and improve sleep initiation and maintenance (Kaplan). Although controlled clinical trials are lacking, this combined chronobiologic approach has been repeatedly reported to substantially improve sleep continuity, reduce daytime sleepiness, and attenuate associated behavioral dysregulation. In one prospective study of 20 children with genetically confirmed SMS, however, melatonin treatment provided limited benefit, improving nocturnal awakening duration, wake-up time, and longest continuous sleep episode on actimetry, but had minimal impact on total sleep time, sleep efficiency, or circadian timing; notably, very high daytime salivary melatonin levels suggested possible iatrogenic accumulation more than 48 hours after discontinuation. β-blockers were associated with a trend toward an earlier melatonin peak but also with delayed sleep onset, increased nocturnal awakenings, and reduced total sleep. Overall, children demonstrated ongoing insomnia, excessive daytime sleepiness, and learning difficulties, indicating that current treatments offer only partial relief and may introduce additional chronobiological complications (Comajuan et al., 2025).

In an online survey directed at caregivers, families consistently described persistent sleep difficulties in patients with SMS, despite structured routines, environmental modifications, melatonin, β-blockers, and behavioral strategies. Caregivers emphasized that these challenges profoundly affected family functioning, contributing to chronic fatigue, emotional strain, and reduced quality of life. To manage nocturnal behaviors and ensure safety, some families relied on protected beds designed to prevent injury during nighttime agitation or wandering as well as limiting nighttime access to certain rooms in the household; however, these solutions were often described as necessary but insufficient. Caregivers also highlighted the critical importance of respite services, noting that short-term relief or overnight support was essential for sustaining caregiving capacity, yet frequently difficult to access. Overall, caregiver experiences underscore that current interventions provide only partial benefit and that effective sleep management in SMS requires not only evidence-based clinical strategies but also practical, safety-oriented supports and accessible respite care for families (Agar et al., 2022).

A subset of SMS individuals may also have sleep-disordered breathing, particularly those with obesity or those taking sedating psychotropic medications; evaluation is necessary when excessive daytime sleepiness persists despite chronotherapy (Kaplan et al., 2020; Tamir et al., 2023).

#### 4.5.2 Angelman syndrome

Angelman syndrome (AS) is a rare neurodevelopmental disorder caused by loss of function of the maternal *UBE3A* gene. It is characterized by motor impairment, intellectual disability, severe speech limitations, and a distinctive behavioral profile marked by frequent laughter and high sociability. Comorbidities are common and include epilepsy, ASD, and significant sleep disturbances. Sleep problems—reported in 20–80% of individuals with AS—include reduced sleep need, abnormal sleep–wake cycles, sleep-onset delay, frequent nighttime awakenings, early morning waking, decreased sleep duration, daytime sleepiness, and overall poor sleep quality (O'Rourke et al., 2024).

Across a systematic review of 10 studies involving 54 individuals with Angelman syndrome, most interventions were pharmacological, with generally mixed effects. Only one study used behavioral treatment alone, consisting of setting a sleep compatible environment, sleep schedule, and recommendations for parent-child interactions with guidance provided via video telehealth/phone contact, and weekly contact with the research team was made. The intervention showed improved increase in independent sleep onset. In the combined study, which consisted of a case study, behavioral interventions consisted of scheduled bedtime, sleep restriction by day, fluid restriction in evening, and returned to bed, associated with a first-generation antihistamine. Sleep duration at night and decrease sleeping by day was achieved (Egan et al., 2020).

In the randomized controlled study conducted by Bindels-de Heus et al. children with Angelman syndrome (AS) and significant sleep problems were assigned either to a structured behavioral sleep intervention or to a control group. The intervention consisted of a standardized program with home visits, parent psychoeducation, direct observation of each child's bedtime routine, individualized feedback based on night-time video recordings, and behavioral techniques delivered by a trained behavioral therapist. Outcomes were measured using sleep diaries, questionnaires, actigraphy, and videosomnography. The intervention led to significant improvements in WASO on videosomnography, and single-case analyses showed additional benefits in total sleep time and WASO based on diaries and actigraphy. These gains were persistent at follow-up, and secondary outcomes demonstrated sustained improvements in sleep hygiene and several parental quality-of-life domains (Bindels-de Heus et al., 2023).

#### 4.5.3 Rett syndrome

Rett syndrome (RTT) is a rare neurodevelopmental disorder caused primarily by *MECP2* mutations, characterized by early developmental regression, loss of hand and communication skills, stereotypies, motor dysfunction, and epilepsy. Sleep disturbances affect over 80% of individuals and may include prolonged sleep latency, frequent night awakenings, nocturnal vocalizations, bruxism, and nighttime seizures. Polysomnography typically shows increased WASO, elevated N3 sleep, reduced REM sleep, and occasional obstructive sleep apnoea. Altered melatonin regulation may contribute to sleep dysfunction, and melatonin supplementation is considered a potential therapeutic approach (Tamir et al., 2023).

In a large cross-sectional study analyzing data from 364 individuals with RTT, over 90% experienced difficulty initiating or maintaining sleep, with frequent night-waking being especially common, producing moderate to major impacts on both children and their families. About three-quarters of participants were not using sleep medications, and melatonin was the most common agent among those treated. Medication use—especially polytherapy—was associated with greater sleep disturbance severity. In contrast, stronger adherence to sleep hygiene practices (regular routines, consistent schedules, appropriate environment) was associated with lower odds of major family impact and better scores on the Disorders of Initiating and Maintaining Sleep scale (Boban et al., 2018).

## 5 Discussion

In this narrative review authors have searched for recent data from systematic reviews, randomized controlled trials, and single-case and cohort studies that have examined sleep assessment and primarily non-pharmacological interventions for sleep problems in children and adolescents with NDDs and some neurological disorders.

Due to the high prevalence of sleep complains/disorders in children with NDD the evaluation of sleep disturbances should follow a structured approach, before interventions start. This evaluation should start with a comprehensive clinical history and physical examination followed by an specific assessment (validated sleep questionnaires, sleep diary) and, when indicated, objective sleep measures, such as actigraphy and/or polysomnography (Bruni et al., 2025).

Behavioral/cognitive interventions should be adapted to age and clinical conditions of the children/adolescent in case. The clinical effectiveness of nonpharmacological interventions is questionable as there are available a limited number of randomized controlled trials. Further, the studies available, shows an heterogeneous nature of the interventions and outcomes used, and the majority has insufficient power to detect effect (Rigney et al., 2018; Bourchtein et al., 2020; Kamara et al., 2025).

Narrative reviews are often useful for topics that require a meaningful synthesis of research evidence that may be complex or broad and that require detailed, nuanced description and interpretation (Sukhera, 2022). However, limitations of this study, as for all narrative reviews, is the recognition of the fact that not all relevant literature on this topic was included.

In conclusion behavioral and educational sleep interventions are generally safe, acceptable, and clinically useful across NDDs, particularly when embedded in multidisciplinary, condition-informed care. However, their efficacy is constrained by small, heterogeneous trials and non-standardized outcome measures.

Future directions should focus on robust, syndrome-specific randomized studies with harmonized sleep and daytime outcomes. Also studies that combine melatonin and behavioral techniques are urgently needed to guide evidence-based practice.
